# A rare case of descending colonic volvulus presenting as large bowel obstruction 19 years after sigmoidectomy and descending colorectal anastomosis

**DOI:** 10.1097/MS9.0000000000001797

**Published:** 2024-02-28

**Authors:** Temesgen Agegnehu Abebe, Yophtahe Woldegerima Berhe, Oumer Ahmed Seid, Worku Mekonnen Sefefe, Leaynadis Kassa Lake

**Affiliations:** a Department of Surgery, School of Medicine, Debre Markos University, East Gojjam; Departments ofb Anesthesia; c Surgery, School of Medicine, University of Gondar, Gondar, Ethiopia

**Keywords:** bowel obstruction, case report, descending colon, extraperitonealization, volvulus

## Abstract

**Introduction and importance:**

Large bowel obstruction is a common surgical emergency worldwide. Large bowel obstruction secondary to descending colonic volvulus is an extremely rare condition with only few reported cases worldwide. Its extreme rarity is due to its retroperitoneal location and lack of mesentery.

**Case presentation:**

A 75-year-old male patient with a history of sigmoidectomy and end-to-end descending colorectal anastomosis 19 years previously, presented with failure to pass faeces and flatus of 1 day duration with associated colicky abdominal pain, distension and vomiting. The abdomen was distended but soft and non-tender. Digital rectal examination showed an empty and ballooned rectum. The intraoperative finding was of a 360° counter-clockwise rotated descending colon. Detorsion and extraperitonealization of the descending colon was performed and the patient was successfully discharged.

**Clinical discussion:**

Volvulus is a twisting of a segment of bowel along its own mesentery. Sigmoid volvulus and caecal volvulus accounts for up to 90% and less than 20% of cases, respectively. Descending colonic volvulus is described in very few case reports. Diagnosis is clinical and confirmed by imaging.

**Conclusion:**

Descending colonic volvulus is a surgical emergency and an extremely rare cause of large bowel obstruction. Surgical management options include extraperitonealization of the descending colon, diversion colostomy or resection and primary end-to-end anastomosis.

## Introduction

Highlights

- Descending colonic volvulus is extremely rare surgical emergency.
- Descending colonic volvulus rarity is due to its retroperitoneal location and lack of mesentery.
- Diagnosis is clinical and confirmed by imaging.
- Patient should be optimized before rushing to surgery.
- Surgical management options include extraperitonealization of the colon, colostomy or resection and anastomosis.

Volvulus occurs when a segment of the bowel twists about its own mesentery. Sigmoid volvulus accounts for up to 90% of cases of large bowel obstruction^1^. Descending colon volvulus is extremely rare because of it’s a secondarily retroperitoneal location and lack of mesentery^2^. There have been very few reports of descending colonic volvulus worldwide. Based on our literature search, this is only the second reported case of descending colon volvulus with a previous history of sigmoidectomy^3^ and the first to be reported in Africa.

We present a case of descending colonic volvulus in a 75-year-old male and the subsequent clinical management.

This case report has been reported in line with the Surgical Case report (SCARE) Criteria^4^.

### Case presentation

A 75-year-old male farmer was brought by his family to the emergency surgical department with a complaint of failure to pass faeces and flatus of 1 day duration, associated with colicky abdominal pain, abdominal distension and two episodes of vomiting which were bilious, non-projectile, foul smelling and non-bloody. Nineteen years previously, he had a laparotomy plus sigmoidectomy with end-to-end descending Colorectal anastomosis for large bowel obstruction secondary to sigmoid volvulus. There had been no associated complications in the intervening time. He has no history of bloody stool, anorexia, weight loss or back pain. He has no history of headache, tinnitus, vertigo or easy fatigability. He has no history of psychiatric illness.

On admission, he looked acutely unwell. His vital signs were in the normal range (blood pressure=130/70 mmHg, PR=64 beats/min, RR=18/min, T=36.8°C, SpO_2_=92% on atmospheric air). He had pink conjunctiva and non-icteric sclera. He had a distended abdomen which moved with respiration. The abdomen was soft, non-tender and hyper-tympanic. There were hyperactive bowel sounds. There was a midline healed surgical scar. Digital rectal examination demonstrated an empty and ballooned rectum with no stool. The prostate was enlarged and firm with findings consistent with asymptomatic benign prostatic hyperplasia. The rest of the physical examination was unremarkable.

**Figure 1:**
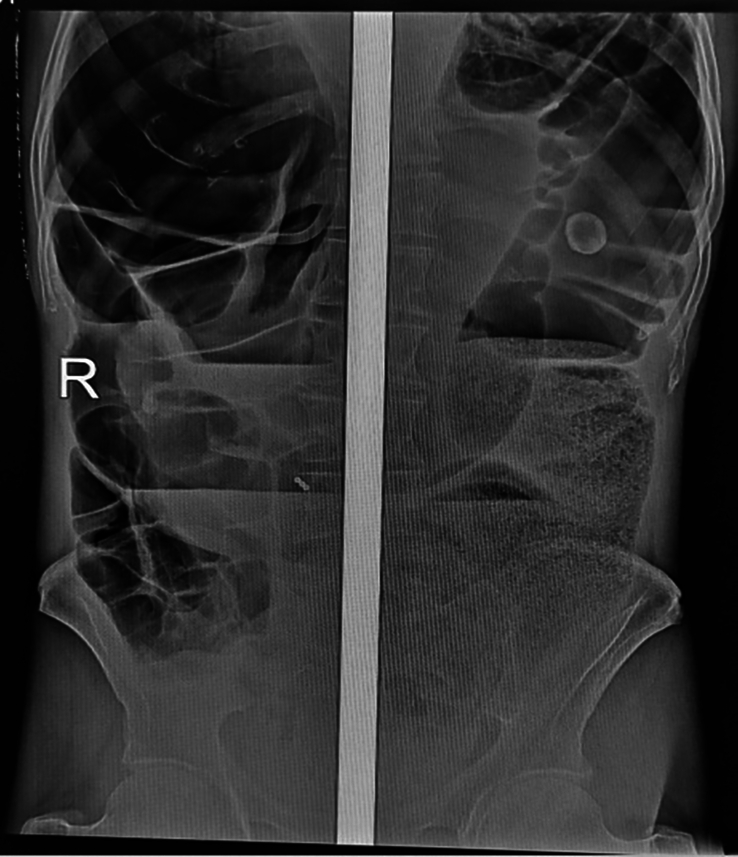
Abdominal X-ray showing a dilated descending colon, transverse colon and caecum loaded with faeces.

White blood cell count was 7.5×10^3^/mm^3^ and showed a left shift with 92% neutrophils and 4.7% lymphocytes. The platelet count was 172×10^3^/mm^3^ and haematocrit was 43%. Other CBC profiles, liver function test, renal function tests and serum electrolytes were in the normal range (Table 1). Erect plain abdominal X-ray showed dilated large bowel loops, paucity of air in the colon and few air-fluid levels suggestive of large bowel obstruction (Fig. 1). A computed tomography (CT) scan was not available in the hospital. On the 1st postoperative day, CBC and serum electrolyte were repeated. WBC count was 7.23 × 10^3^/mm^3^ and showed 68% neutrophils and 24.4% lymphocytes. Hematocrit was 33.9%. the serum electrolytes were in the normal range (Table 1).

**Table 1:** Laboratory investigations of the patient on the day of admission and on the 1^st^ postoperative day

Laboratory Investigations					
On the day of admission (preoperatively)			On the 1^st^ postoperative day		
Complete Blood Cell (CBC) Count					
White Blood Cell	WBC	7.5×10^3^/mm^3^	White Blood Cell	WBC	7.23×10^3^/mm^3^
Red Blood Cell	RBC	4.76×10^6^/mm^3^	Red Blood Cell	RBC	3.81×10^6^/mm^3^
Hemoglobin	HGB	15.2g/dL	Hemoglobin	HGB	11.7g/dL
Hematocrit	HCT	43%	Hematocrit	HCT	33.9%
Neutrophil	NEUT	92.7%	Neutrophil	NEUT	68%
Lymphocyte	LYMP	4.7%	Lymphocyte	LYMP	24.4%
Eosinophil	EOS	0.0%	Eosinophil	EOS	4.1%
Monocyte	MONO	2.5%	Monocyte	MONO	3%
Basophil	BASO	0.1%	Basophil	BASO	0.5%
Mean Corpuscular Hemoglobin	MCH	31.9pg/cell	Mean Corpuscular Hemoglobin	MCH	30.8 pg/cell
Mean Corpuscular Volume	MCV	90.3 fL	Mean Cell Volume	MCV	88.8 fL
Mean Corpuscular Hemoglobin Concentration	MCHC	35.4 g/dL	Mean Corpuscular Hemoglobin Concentration	MCHC	34.7 g/dL
Red Blood Cell Distribution Width	RDW	14.3 %	Red Blood Cell Distribution Width	RDW	42.2 %
Platelet Count	PLT	172×10^3^/mm^3^	Platelet Count	PLT	167×10^3^/mm^3^
Mean Platelet Volume	MPV	9.8fL	Mean Platelet Volume	MPV	6.9 fL
Liver and renal function tests					
Aspartate Transaminase	SGOT	46 U/L			
Alanine Transaminase	SGPT	39 U/L			
Serum Albumin	ALB	3.9 g/dL			
Bilirubin, Direct	BILI D	0.28 mg/dL			
Bilirubin, Total	BILI T	1.5 mg/dL			
Blood Urea Nitrogen	BUN	12 mg/dL			
Serum Creatinine	CREAT	0.51 mg/dL			
Alkaline Phosphatase	ALP	82 IU/L			
Serum Electrolytes					
Sodium	Na	135.9 mEq/L			142 mEq/L
Potassium	K	4.17 mmol/L			3.8 mmol/L
Chloride	Cl	104 mEq/L			98 mEq/L
Calcium, Ionized	Ca	2.2 mmol/L			2.3 mmol/L

**Figure 2:**
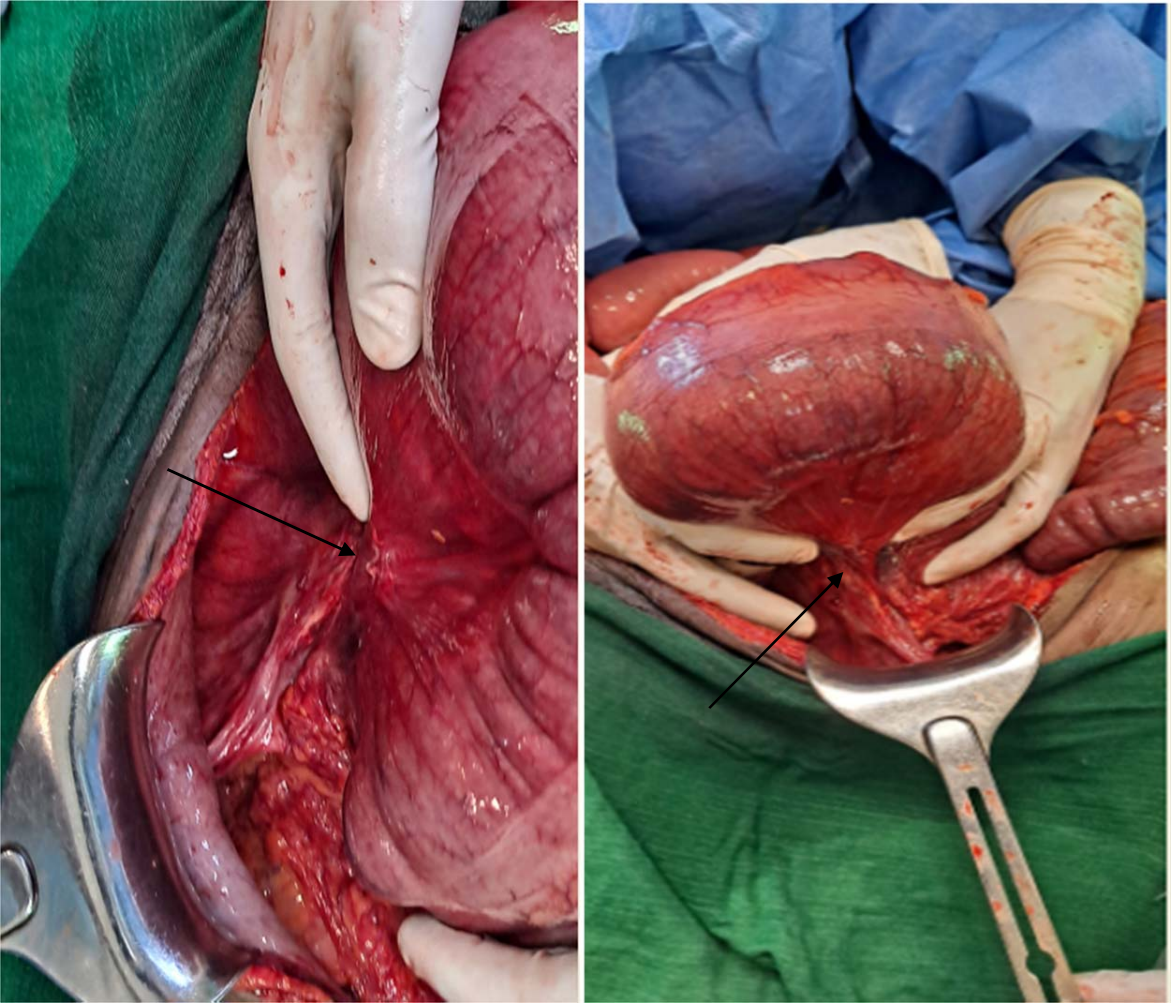
Intraoperative view demonstrating volvulus of descending colon and mesenteric twisting site (black arrow) without signs of ischaemia.

**Figure 3:**
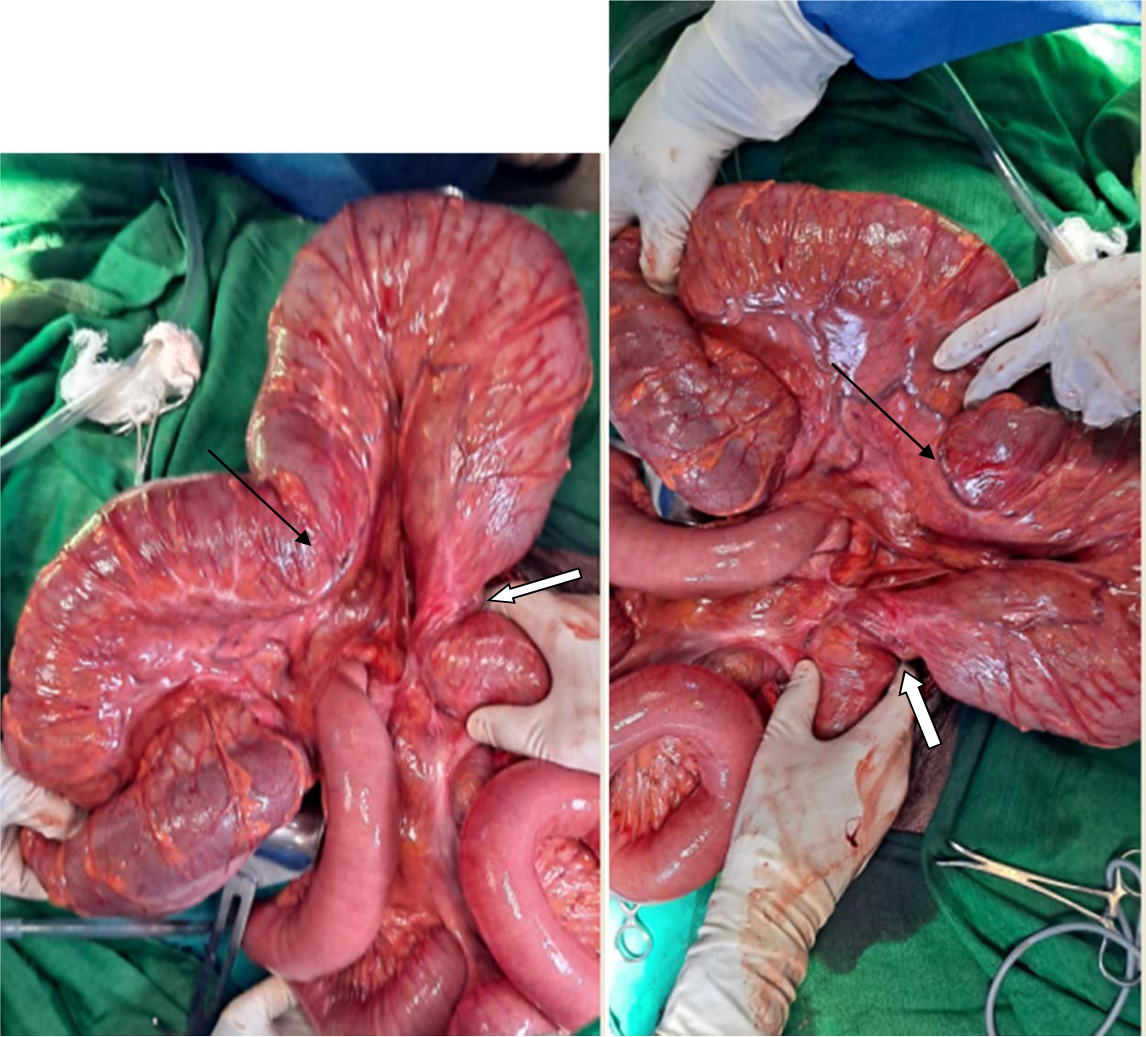
Intraoperative view demonstrating dilated transverse colon, descending colon, splenic flexure (black arrow) and previous descending Colorectal anastomotic site (white arrow).

**Figure 4:**
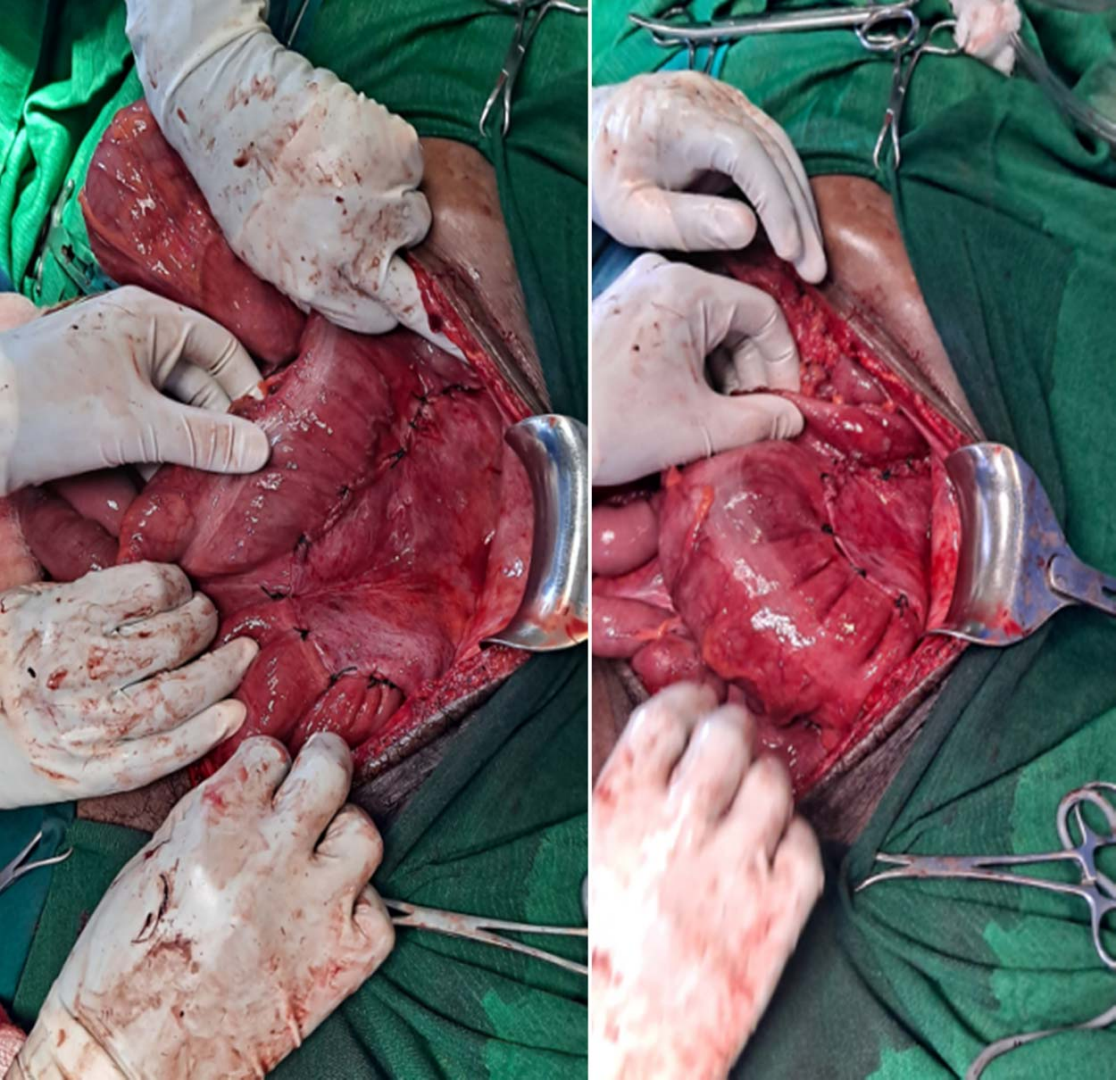
Intraoperative view demonstrating extraperitonealization of descending colon by fixing lateral coli of descending colon with lateral peritoneal reflection of abdominal wall.

Initial management included IV Ringer lactate as a 1L bolus followed by maintenance infusion. Deflation with a rectal tube was not successful. During subsequent exploratory laparotomy, the intraoperative finding was 360° counter-clockwise rotated descending colon also involving part of distal transverse colon and splenic flexure (Fig. 2). The descending colon was entirely intraperitoneal. The caecum, ascending colon, hepatic flexure, transverse colon, splenic flexure and descending colon were significantly dilated (Fig. 3). The previous descending Colorectal anastomosis site was intact and minimally constricted (Fig. 3). The colonic volvulus was derotated and deflated with a rectal tube intraoperatively. Then extraperitonealization of the descending colon was done by fixing the lateral coli of the descending colon with the lateral peritoneal reflection of the abdominal wall (Fig. 4). Postoperative recovery was smooth and the patient was discharged after 3 days of hospital stay. On his 12th postoperative day, he was examined at the surgical referral clinic and he was able to tolerate oral feeds, with no abdominal pain, distension, fever, vomiting or constipation. Further out-patient follow-up done at 4 weeks and 6 months, following the surgery, the patient remained stable and he had resumed normal activities.

## Discussion

The descending colon is a secondarily retroperitoneal organ. During development, the primitive dorsal mesocolon fuses with parietal peritoneum at 5–6 months of gestation.

Volvulus is a twisting of a section of bowel around its own mesentery. In western countries, colonic volvulus is the third leading cause of colonic obstruction accounting less than 5% of all large bowel obstructions, behind colorectal cancer and diverticular disease^3,5^. However, in low-income countries volvulus is the most common cause of large bowel obstruction. In the “volvulus belt” that extends through South America, Africa, the Middle East, India and Russia, colonic volvulus accounts about 50% of all cases of colonic obstruction. This higher incidence is attributed to a high fibre and vegetable diet^5,6^. Other risk factors attributed for large bowel obstruction include advanced age, chronic constipation, medications affecting colonic motility, history of abdominal surgery, neurologic or psychiatric abnormalities and megacolon^5,7^. In Ethiopia, sigmoid volvulus alone accounts greater than 54% of all intestinal obstruction. Sigmoid volvulus and caecal volvulus account for up to 90% and less than 20% of volvulus, respectively^1^. The prevalence of sigmoid volvulus varies significantly ranging from 1 to 7% in the United States, to 56% in Ethiopia as high as 80% in the Andes^8–10^. The transverse colon and splenic flexure are rarely involved, accounting for less than 5% of cases^11^. Descending colonic volvulus as a cause of large bowel obstruction is extremely rare. This is the first case of descending colonic volvulus after sigmoidectomy to be reported in Africa.

Clinical presentation of large bowel obstruction is a failure to pass faeces and/or flatus, colicky abdominal pain, abdominal distension and less frequent vomiting, typically feculent. Proximal obstruction results in less abdominal distention and more frequent vomiting. Conversely, more distal obstruction results in significant distension and less frequent vomiting. When the obstruction becomes gangrenous, for example due to delayed presentation, the patient will develop peritonitis with a toxic presentation such as persistent abdominal pain, fever, hypothermia, tachycardia, hypotension and tachypnea. Such a patient will have abdominal tenderness and rigidity. Digital rectal examination may demonstrate an empty ballooned rectum, sometimes with tenderness and blood^1,5,6^. Our patient had all the cardinal features of large bowel obstruction but without toxic features.

Imaging studies usually confirm the diagnosis of bowel obstruction. Large bowel obstruction in an erect plain abdominal X-ray may show dilated peripherally located colon greater than 6 cm, air-fluid levels and a paucity of air in the colon^1,5,6^. CT scan and contrast enema may be obtained if needed to look for “coffee bean” sign, “whirl” sign or “birds’ beak” appearance^1,3,5^. Plain abdominal x-ray ordered and it was consistent with findings of large bowel obstruction (Fig. 1). The sensitivity, specificity and accuracy of plain abdominal X-ray in diagnosis the presence of intestinal obstruction is 77%, 50% and 75%, respectively. Compared to plain abdominal X-ray, CT scan has higher sensitivity, specificity and accuracy of 93%, 100% and 94%%, respectively^12^. However, CT scan was not available in our hospital at that time and, most importantly, such advanced imaging studies are not necessary to establish the diagnosis^6^.

For simple large bowel obstruction, urgent intervention is necessary to prevent development of necrosis, perforation and peritonitis. The patient should be optimized with fluid resuscitation and correction of electrolytes. The timing and choice of definitive surgical management depends on the patient condition and presentation^5^. After resuscitation and patient optimization, decompression is recommended with sigmoidoscope and placement of a rectal tube. Then, resection and anastomosis can be done later, to reduce morbidity and mortality associated with emergency surgery. Non-operative decompression with rectal tube deflation is contraindicated if there are signs and symptoms of peritonitis due to bowel ischaemia and necrosis. If detorsion and deflation with rectal tube is unsuccessful, or contraindicated, then emergency surgery is required. If there are signs of peritonitis due to colonic ischaemia, necrosis or perforation, traditionally an end-colostomy (Hartmann’s procedure) is performed. However, there is now increasing support for resection with primary anastomosis^6^.

A similar case is reported of descending colonic volvulus with a previous sigmoidectomy in Mercy Hospital, St Louis, MO, USA. This was managed with a two-stage procedure. Firstly, an endoscopic decompression (deflation) with flexible sigmoidoscopy was performed and then, two days’ later, a laparoscopic anterior colon resection including resection of the previous colorectal anastomosis was done^13^. Although such a minimally invasive approach is better tolerated by the patient, there is additional risk of anastomotic leak, since they did resection and anastomosis. Shaposhnikov *et al.* ^11^ recommends keeping the rectal tube in situ and doing elective surgery within 48 hours of detorsion and deflation. In our practice, following successful deflation with a rectal tube, the definitive resection and end-to-end anastomosis is usually done about 2 weeks later, to allow bowel oedema resolution and to minimize complications of doing surgery on an unprepared bowel. Conversely, some literature recommends doing resection and primary end-to-end anastomosis even for gangrenous large bowel obstruction on an emergency basis^14^. Interestingly, recent work suggests there is no significant difference between primary anastomosis after resection of gangrenous versus viable bowel in terms of hospital stay, rate of anastomotic leak or mortality^15^. The approach at Mercy Hospital was different from our approach, because we did laparotomy with an infraumbilical incision and extraperitonealization of the colon while they performed sigmoidoscopic deflation followed by laparoscopic resection and primary anastomosis^13^. Lack of laparoscopic service at duty hours in our hospital can be considered as a limitation on the patient management part. Since laparoscope is a minimally access surgery, it offers advantages of small incision, minimal blood loss, early postoperative ambulation, less postoperative pain and early discharge from the hospital^16^.

In another case report of descending colonic volvulus, successful deflation was done via a colonoscope but the patient demanded the rectal tube be removed immediately, due to discomfort, and surgery with an end-colostomy was done and the patient died after 2 days. The mortality rate for descending colonic volvulus is not known because of the few case reports, but for sigmoid, caecal and transverse colon volvulus was it is quoted as 9.44%, 6.64% and 18%, respectively^11^.

Limitation of this study is inability to determine cause-effect relationship and lack of ability to generalize although it detected this extremely rare clinical condition and offers high educational value^17^.

## Conclusion

Descending colonic volvulus is an extremely rare cause of large bowel obstruction due to its retroperitoneal location and lack of mesentery. Early diagnosis and prompt management with endoscopic decompression is recommended if there are no symptoms and signs of peritonitis. If deflation is unsuccessful or if there are symptoms and signs of peritonitis from the outset, emergency surgery is indicated. The surgical options include extraperitonealization, diversion colostomy or resection and primary end-to-end anastomosis, depending on patient co-morbidity, hemodynamic condition, nutritional status and the operator.

## Ethical clearance

Ethical approval for publication of this case report was provided by the Ethical Review Board of School of Medicine on 23 May 2023. IRB file number is 772/2015.

## Consent

Written informed consent was obtained from the patient for publication of this case report and the images. A copy of the written consent is available for review by the Editor-in-Chief of this journal on request.

## Source of funding

This case report did not receive any specific grant from funding agencies in the public, commercial, or not-for-profit sectors.

## Author contribution

T.A.A. led the manuscript preparation. T.A.A., O.A.S., L.K.L. and Y.W.B. operated and managed the patient in the hospital. Y.W.B. and W.M.S. criticized the manuscript. All authors contributed to the writing and editing of this case report. All authors read and approved the final manuscript. All authors have agreed to be accountable for all aspects of this report.

## Conflicts of interest disclosure

No potential conflict of interest relevant to this article was reported.

## Availability of data and materials

The authors of this manuscript are willing to provide any additional information regarding the case report.

## Provenance and peer review

Not commissioned, externally peer-reviewed.
